# Editorial: Management of congenital heart disease: challenges, implications, innovations and pitfalls

**DOI:** 10.3389/fped.2025.1599311

**Published:** 2025-07-01

**Authors:** Stefano Carmelo Di Bernardo

**Affiliations:** Department of Pediatrics/Pediatrics Cardiology Unit, Centre Hospitalier Universitaire Vaudois (CHUV), Lausanne, Switzerland

**Keywords:** congenital heart disease, challenges, genetics, intervention, imaging

**Editorial on the Research Topic**
Management of congenital heart disease: challenges, implications, innovations and pitfalls

## Introduction

Congenital heart disease (CHD) poses one of the most complex challenges in modern medicine. Impacting numerous people worldwide, it affects individuals from fetal development through adulthood, necessitating precise diagnosis along with personalized and adaptive treatment strategies. Congenital heart disease care is rapidly evolving, driven by advancements in molecular biology, imaging technologies, intervention techniques, and patient monitoring. This research topic features eight contributions that highlight this evolution. Instead of presenting these advancements in isolation, I aimed to connect these works and create a compelling narrative for the future management of CHD.

### From rare mutations to physiological adaptation: refining our understanding

Genetic and developmental biology advancements are crucial to decoding the diverse phenotypes seen in CHD. In a striking case report, Deng et al. describe a restrictive cardiomyopathy caused by a rare TNNI3 mutation, highlighting the diagnostic synergy of cardiac MRI and next-generation sequencing. Such reports underscore the power of personalized molecular insights to guide management in unexplained or severe presentations.

Physiological adaptation is another dimension of complexity in CHD. In their systematic review and meta-analysis, Vecchiato et al. explore how patients with CHD perform during cardiopulmonary exercise testing at high altitudes. Their findings demonstrate a significantly reduced exercise capacity compared to sea-level peers—a reminder that geography and oxygen availability are hidden yet potent modifiers of clinical outcomes.

Similarly, McCrary et al. describe electromechanical dyssynchrony of the right ventricle after tetralogy of Fallot repair in infants. This finding has implications for long-term surveillance and may influence postoperative pacing strategies or imaging follow-up.

### Improving interventions: toward less invasive and more patient-centered care

In CHD, interventions are often staged and technically complex. However, they are also rapidly evolving. Prakoso et al. compare ductal stenting to surgical shunting in patients with late-presenting, duct-dependent pulmonary circulation. Their results—showing reduced hospital stays and mortality—reinforce the potential of catheter-based approaches even in traditionally reserved surgery settings.

Building on this, Rosenthal et al. report on an application-based interstage home monitoring (IHM) program for infants with shunt- or duct-dependent pulmonary perfusion. Their success in reducing interstage mortality through digital tools speaks to a future where connected care and parental empowerment become cornerstones of CHD management.

Meanwhile, Ghoussaini et al. provide a retrospective analysis of pulmonary artery banding in a tertiary center that spans two decades. Their insights into indications, outcomes, and complications help refine our understanding of this palliative approach, particularly in low-resource or high-complexity contexts.

### Connecting metabolism, anatomy, and imaging: toward precision monitoring

Metabolomics is a growing field with immense potential in pediatric cardiac care. Meggiolaro et al. review the existing literature on metabolomic profiles in infants undergoing cardiopulmonary bypass, linking metabolic patterns to outcomes like acute kidney injury and neurologic complications. These emerging biomarkers may enable real-time risk stratification and individualized organ protection strategies.

Anatomic variation also plays a pivotal role in outcomes. In their report of two cases and a literature review, Yang et al. detail anomalous pulmonary venous return due to septum primum malposition—a subtle but significant lesion. Their findings advocate for meticulous echocardiographic assessment in infants with unexplained desaturation or right-sided volume overload.

## Conclusion

These eight contributions collectively reflect the breadth and depth of current advances in congenital heart disease. Each article reinforces the need for an integrated, patient-centered approach, from molecular diagnosis to long-term functional outcomes and surgical technique to digital follow-up. As CHD care evolves, such multidisciplinary insights will be essential to building a future of truly individualized and adaptive medicine.

